# Acetylated Diacylglycerol 1-palmitoyl-2-linoleoyl-3-acetyl-rac-glycerol in Autoimmune Arthritis and Interstitial Lung Disease in SKG Mice

**DOI:** 10.3390/biomedicines9091095

**Published:** 2021-08-27

**Authors:** Doo-Ho Lim, Eun-Ju Lee, Hee-Seop Lee, Do Hoon Kim, Jae-Hyun Lee, Mi Ryeong Jeong, Seokchan Hong, Chang-Keun Lee, Bin Yoo, Jeehee Youn, Yong-Gil Kim

**Affiliations:** 1 Division of Rheumatology, Department of Internal Medicine, Ulsan University Hospital, University of Ulsan College of Medicine, Ulsan 44033, Korea; dlaengh@hanmail.net; 2 Asan Institute for Life Science, Asan Medical Center, Seoul 05505, Korea; krys72@hanmail.net; 3 Department of Nutrition and Food Science, College of Agriculture and Natural Resources, University of Maryland, College Park, MD 20742, USA; hslee123@umd.edu; 4 Division of Rheumatology, Department of Internal Medicine, Asan Medical Center, University of Ulsan College of Medicine, Seoul 05505, Korea; alakzam41@naver.com (D.H.K.); clesote@hanmail.net (J.-H.L.); mi-ryeong@naver.com (M.R.J.); medivineluke@gmail.com (S.H.); cklee@amc.seoul.kr (C.-K.L.); byoo@amc.seoul.kr (B.Y.); 5 Department of Anatomy and Cell Biology, College of Medicine, Hanyang University, Seoul 04763, Korea; jhyoun@hanyang.ac.kr; 6 Convergence Medicine Research Center, Asan Institute for Life Science, Asan Medical Center, Seoul 05505, Korea

**Keywords:** interstitial lung disease, PLAG, NETosis, arthritis, autoimmune

## Abstract

Acetylated diacylglycerol 1-palmitoyl-2-linoleoyl-3-acetyl-rac-glycerol (PLAG) is a lipid molecule from the antlers of sika deer that might reduce inflammation by effectively controlling neutrophil infiltration, endothelial permeability and inflammatory chemokine production. Therefore, we evaluated the modulatory effect of PLAG on arthritis and interstitial lung disease (ILD) in an autoimmune arthritis model. We injected curdlan into SKG mice and PLAG was orally administered every day from 3 weeks to 20 weeks after the curdlan injection. The arthritis score was measured every week after the curdlan injection. At 20 weeks post-injection, the lung specimens were evaluated with H&E, Masson’s trichrome and multiplexed immunofluorescent staining. Serum cytokines were also analyzed using a Luminex multiple cytokine assay. PLAG administration decreased the arthritis score until 8 weeks after the curdlan injection. However, the effect was not sustained thereafter. A lung histology revealed severe inflammation and fibrosis in the curdlan-induced SKG mice, which was attenuated in the PLAG-treated mice. Furthermore, immunofluorescent staining of the lung tissue showed a GM-CSF^+^ neutrophil accumulation and a decreased citrullinated histone 3 expression after PLAG treatment. PLAG also downregulated the levels of IL-6 and TNF-α and upregulated the level of sIL-7Rα, an anti-fibrotic molecule. Our results indicate that PLAG might have a preventative effect on ILD development through the resolution of NETosis in the lung.

## 1. Introduction

Interstitial lung disease (ILD) is one of the most serious pulmonary complications in autoimmune diseases and is a major cause of morbidity and mortality [1]. In general, the five major autoimmune diseases with ILD are known as systemic sclerosis, rheumatoid arthritis (RA), polymyositis-dermatomyositis, systemic lupus erythematosus and primary Sjögren syndrome [1]. Among these, RA is the most common autoimmune disease and affects approximately 1% of the population [2] and is characterized by systemic inflammation resulting in peripheral arthritis and various pulmonary manifestations [3]. The etiology of RA is not known but complex interactions between genetic backgrounds and environmental factors such as smoking and infection might cause a breakdown of the immune regulation and inflammatory mechanisms leading to systemic inflammation and tissue damage [4]. Although RA primarily affects the peripheral joints, up to 30% of RA patients develop RA-associated ILD [5,6], a serious diffuse parenchymal lung disease related to inflammation and fibrosis. This disease is the main cause of morbidity and mortality among RA patients [7]. However, there are limited data about the treatment of RA-associated ILD.

T cells play an important role in the immune response in RA. Autoreactive T cells infiltrate the synovial tissue and promote an adaptive immune response, resulting in the overproduction of pro-inflammatory cytokines such as tumor necrosis factor alpha (TNF-α), interleukin (IL)-6, IL-17 and interferon gamma (IFN-γ) [8]. Inflammatory arthritis in RA patients has been improved by the use of conventional and biological disease modifying anti-rheumatic drugs (DMARDs) that regulate the immune cells and pro-inflammatory cytokines related to the disease. However, several reports show an association between DMARDs and adverse respiratory events, suggesting that DMARDs exacerbate pulmonary dysfunction despite beneficial effects on joint disease [9,10,11]. In addition to autoreactive T cells, neutrophils are present in the lung tissue and have a key role in RA-associated ILD [12,13,14,15]. Patients with RA-associated ILD show an association between neutrophilia in the bronchoalveolar lavage fluid and advanced interstitial fibrosis, suggesting a potential role of human neutrophil elastase and myeloperoxidase activity or neutrophil-derived oxidative injury [16]. Moreover, a previous report described that excessive neutrophil extracellular traps (NETs), a complex of DNA and neutrophil-derived antimicrobial molecules, might be related to lung inflammation and fibrosis in connective tissue disease-associated ILD [17]. Therefore, neutrophils and associated inflammatory cascades might be possible therapeutic targets in RA-associated ILD.

Acetylated diacylglycerol 1-palmitoyl-2-linoleoyl-3-acetyl-rac-glycerol (PLAG) is a lipid molecule that has been isolated from the antlers of sika deer [18]. PLAG can be chemically synthesized as a single compound from glycerol, palmitic acid and linoleic acid and is confirmed to be identical to the naturally isolated form [19]. In previous studies, PLAG improved inflammation and joint destruction by reducing neutrophil infiltration into the synovial joints in collagen-induced arthritis mice [20]. Further, PLAG ameliorated a lung injury through the effective control of neutrophil infiltration, endothelial permeability and inflammatory chemokine production [21]. Thus, PLAG may be a potential therapeutic agent for autoimmune arthritis and associated ILD. 

SKG mice are an animal model in which chronic autoimmune inflammatory arthritis is developed following exposure to beta-glucan. These mice have a mutation in the gene encoding of the SH2 domain of ZAP-70, a key signal transduction molecule in T cells. This mutation results in a failure in the negative selection of highly self-reactive T cells [22]. We previously showed that after a curdlan injection, SKG mice develop lung inflammation, fibrosis, arthritis and lung lesions [13]. Thus, this mouse might be a good candidate for a murine autoimmune arthritis and associated ILD model. Therefore, we investigated the effect of PLAG treatment on peripheral arthritis and ILD development in this model. 

## 2. Materials and Methods

### 2.1. Induction of Arthritis and Interstitial Lung Disease in SKG Mice

**Figure 1:**
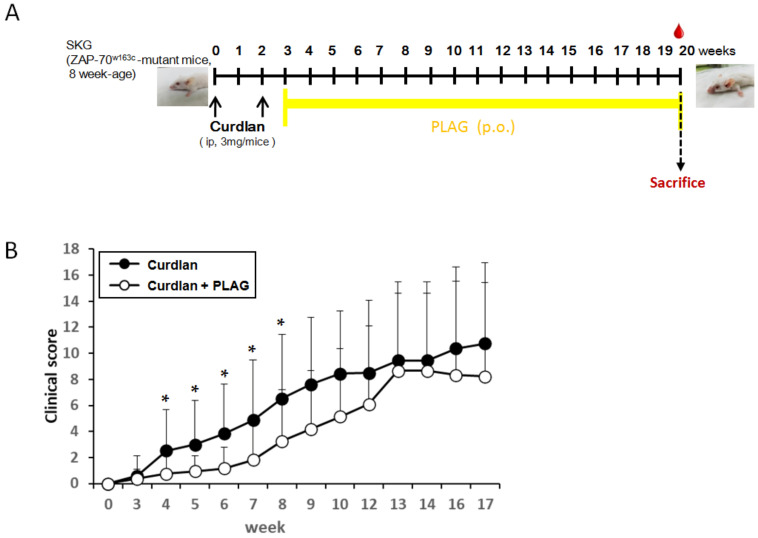
**Experimental design and the clinical score of peripheral arthritis.** (**A**) Experimental design. Eight-week-old male SKG mice were intraperitoneally injected with 3 mg curdlan at 0 and 2 weeks. The arthritis scores were measured every week for up to 17 weeks. In half of the curdlan-induced SKG mice, PLAG was orally administered from 3 weeks to 20 weeks after the curdlan injection. We identified the presence of ILD by a histological analysis at 20 weeks post-injection. (**B**) The clinical score of peripheral arthritis. The arthritis scores were significantly lower in the PLAG-treated curdlan-induced SKG mice, beginning 1 week after PLAG administration. The reduced scores continued significantly until 8 weeks after the first curdlan injection. The data are indicated as a mean ± SD; * *p* < 0.05. ip: intraperitoneal injection; ILD: interstitial lung disease; PLAG: acetylated diacylglycerol 1-palmitoyl-2-linoleoyl-3-acetyl-rac-glycerol.

SKG mice were obtained from Dr. S. Sakaguchi [23]. As shown in Figure 1A, 8-week-old male SKG mice were intraperitoneally injected with 3 mg curdlan suspended in 0.2 mL PBS (*n* = 15) or 0.2 mL PBS alone (control; *n* = 5) at 0 and 2 weeks. We identified the presence of ILD by a histological analysis at 20 weeks post-injection. Arthritis scores were measured every week for up to 17 weeks. In half of the curdlan-induced SKG mice (*n* = 8), PLAG (250 mg/kg body weight/day, Enzychem Lifesciences Co., Daejeon, Korea) was orally administered every day from 3 weeks to 20 weeks after a curdlan (Fuji Wako Pure Chemical Corp, Osaka, Japan) injection. All mice experiments were performed in accordance with the guidelines for animal care approved by the Animal Experimentation Committee of the Asan Institute for Life Sciences (2018-11-172).

### 2.2. Clinical Scoring of Peripheral Arthritis

The clinical features of the peripheral joints were monitored weekly and scored for severity on a scale of 0–4 (0 = no swelling, 1 = mild swelling and redness on the top of the foot, 2 = severe swelling and redness on the top of the foot, 3 = severe swelling and redness on the wrist or ankle joints and 4 = severe swelling of the wrist or ankle joints and digits) [24]. The scores of the affected joints were summed.

### 2.3. Lung Histology

At 20 weeks post-injection, the mice were euthanized by carbon dioxide inhalation and the lung was perfused with PBS via the heart to remove bronchoalveolar and blood cells. The lung was inflated with 10% buffered formalin, embedded in paraffin and sectioned at 4 µm thickness. The sections were then stained with hematoxylin and eosin (H&E) and Masson’s trichrome. The severity of the lung fibrosis was assessed according to the scale by Ashcroft et al. [25] as follows: 0 = normal lung, 1 = minimal fibrous thickening of the alveolar or bronchiolar walls, 3 = moderate thickening of the walls without obvious damage to the lung architecture, 5 = increased fibrosis with definite damage to the lung structure and the formation of fibrous bands or small fibrous masses, 7 = severe distortion of the structure and large fibrous areas (a honeycomb lung was placed in this category), 8 = total fibrous obliteration of the field.

### 2.4. Analysis of the Serum Cytokines by a Luminex Multiplex Cytokine Assay

Serum cytokines were analyzed using a Luminex multiplex cytokine assay at 20 weeks post-injection. The serum samples were prepared by allowing the blood to clot for at least 1 h at RT. The samples were then centrifuged at 16,000× *g* for 15 min at 4 °C. The serum concentrations of the following immune molecules were determined using a magnetic bead-based 8-plex immunoassay: eotaxin, vascular endothelial growth factor (VGEF), IFN-γ, IL-6, IL-10, soluble IL-7Rα (sIL7Rα), IL-22 and TNF-α (customized Procartaplex, Thermo Fisher Scientific, Waltham, MA, USA). Briefly, the serum samples were mixed with antibody-linked polystyrene beads in 96-well filter bottom plates and incubated at RT for 2 h on an orbital shaker at 500 rpm. After washing, the plates were incubated with a biotinylated detection antibody for 30 min at RT. The plates were then washed twice and resuspended in streptavidin-PE. After incubation for 30 min at RT, two additional washes were performed and the samples were resuspended in a reading buffer. Each sample was measured in duplicate along with standards (7-point dilutions) and buffer-only controls. The plates were read using a Luminex Bio-plex 200 system (Bio-Rad Corp., Hercules, CA, USA).

### 2.5. Immunofluorescent Staining

Multiplexed immunofluorescent staining of the lung tissue was performed using the Opal method (Perkin Elmer, Waltham, MA, USA). Primary antibodies were sequentially applied to a single slide. The slides were deparaffinized in xylene and rehydrated in ethanol. Antigen retrieval was performed by heating the slides in a citrate buffer (pH 6.0) using a microwave. Primary rabbit antibodies to neutrophils (Abcam, Cambridge, MA, USA, ab2557, 1:200) were incubated for 1 h in a humidified chamber at RT followed by a detection using polymer horseradish peroxidase (HRP). Mouse (Ms) + Rabbit (Rb) neutrophils were visualized using fluorescein Opal 520 (1:100), after which the slide was placed in a citrate buffer (pH 6.0) and heated using a microwave. In a serial fashion, the slides were then incubated with primary rabbit antibodies to a granulocyte-macrophage colony-stimulating factor (GM-CSF) (Abcam, Cambridge, MA, USA, ab9741, 1:200) for 1 h in a humidified chamber at RT followed by a detection using polymer HRP Ms + Rb. The GM-CSF was visualized using Opal 620 (1:100). The slides were again placed in a citrate buffer (pH 6.0), heated in a microwave and incubated with primary rabbit antibodies for IL-17A (Novus Biologicals, Centennial, CO, USA, NBP1-76337, 1:500) for 1 h in a humidified chamber at RT followed by a detection using polymer HRP Ms + Rb. IL-17A was visualized using Opal 690 (1:100). Finally, the slides were again placed in a citrate buffer (pH 6.0) and heated using a microwave. The nuclei were visualized with DAPI (1:500) and the sections were mounted using mounting media (HIGHDEF^®^ ADI-950-260-0025, Enzo Life Sciences). The stained slides were scanned with a multispectral Vectra scanner and quantitative imaging system (Perkin Elmer). To observe NETosis in the lung tissues, the slides were incubated with rabbit anti-citrullinated histone 3 (Abcam, Cambridge, MA, USA, ab5103, 1:200) and rat anti-neutrophil (1:50). The slides were then incubated with Opal 540 for neutrophils and Opal 690 for citrullinated histone 3. Other procedures were identical as described above.

### 2.6. Statistical Analysis

All analyses were performed using GraphPad Prism 5 software (GraphPad Software, San Diego, CA, USA). Mann–Whitney U tests were performed for two-group comparisons. *p* < 0.05 was considered statistically significant. The error bars in all figures indicate the standard error (SEM).

## 3. Results

### 3.1. Effect of PLAG on Arthritis

Within 3 weeks after the first curdlan injection, SKG mice developed inflammatory arthritis with swelling and redness in peripheral joints as previously reported [22]. The PBS-injected SKG mice did not show any features of joint inflammation. The effect of PLAG on peripheral arthritis in the curdlan-induced SKG mice was examined weekly. The arthritis scores were significantly lower in the PLAG-treated mice, beginning at 1 week after PLAG administration. The reduced scores observed in these mice continued until 8 weeks after the first curdlan injection (Figure 1B). A statistical significance was not observed after 9 weeks although the score seemed to decrease during the observation period.

### 3.2. Effect of PLAG on Lung Inflammation and Fibrosis

**Figure 2:**
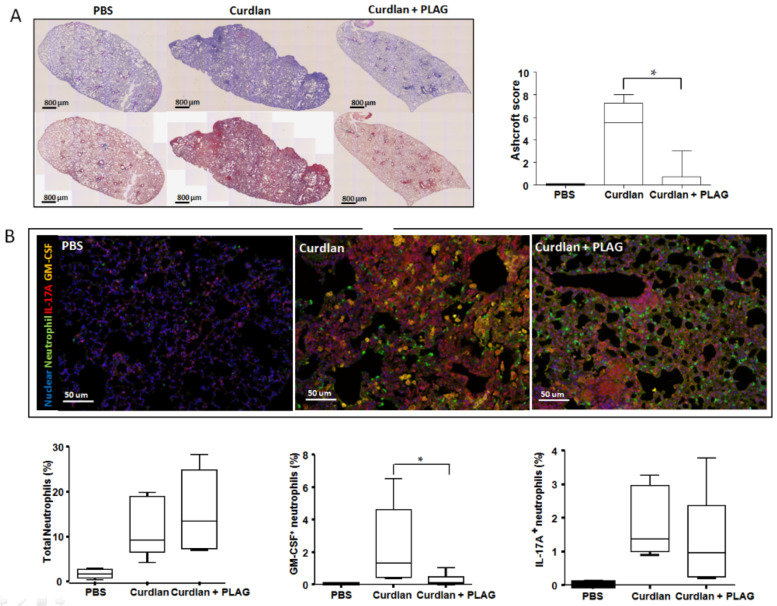
**Histologic examination of the lung tissue from SKG mice at 20 weeks after a curdlan injection with/without PLAG treatment.** (**A**) Hematoxylin and eosin (upper panels) and Masson’s trichrome (lower panels) staining of the lung tissues. Severe lung inflammations were observed in the curdlan-induced SKG mice and these features were attenuated in the PLAG-treated curdlan-induced SKG mice. (**B**) Opal multiplexed immunofluorescent images and semi-quantitation of the total, GM-CSF^+^ and IL-17A^+^ neutrophil accumulation in the lung tissues. The GM-CSF^+^ neutrophil accumulation was significantly decreased in the PLAG-treated curdlan-induced SKG mice compared with those without PLAG treatment whereas the IL-17A^+^ neutrophil accumulation was not attenuated. The values represent the mean of three independent experiments ± SEM. * *p* < 0.05. PLAG: acetylated diacylglycerol 1-palmitoyl-2-linoleoyl-3-acetyl-rac-glycerol.

Lung specimens were evaluated at 20 weeks after the first curdlan injection. Severe lung inflammation, including bronchial alveolar tissue damage and fibrosis, was observed in the curdlan-induced SKG mice but not in the PBS-injected SKG mice (Figure 2A). These features were attenuated in the PLAG-treated curdlan-induced SKG mice. The Ashcroft score of the lung tissue showed significantly lower lung fibrosis in the curdlan-induced SKG mice treated with PLAG compared with the curdlan-induced SKG mice without PLAG treatment (Figure 2A).

In a previous report [13], GM-CSF^+^IL-17A^+^ neutrophils were reported to be the major infiltrating cells in ILD of the curdlan-induced SKG mice. Thus, to observe the co-localization of neutrophils and cytokines after PLAG treatment, an Opal multiplexed immunofluorescent staining examination was also performed in the lung tissues. There was no difference in the total neutrophil accumulation in the curdlan-induced SKG mice regardless of PLAG treatment (Figure 2B). However, the GM-CSF^+^ neutrophil accumulation was significantly decreased in the curdlan-induced SKG mice treated with PLAG compared with those without PLAG treatment. Additionally, the IL-17A^+^ neutrophil accumulation was not attenuated (Figure 2B).

### 3.3. Changes of Serum Cytokines after PLAG Treatment

**Figure 3:**
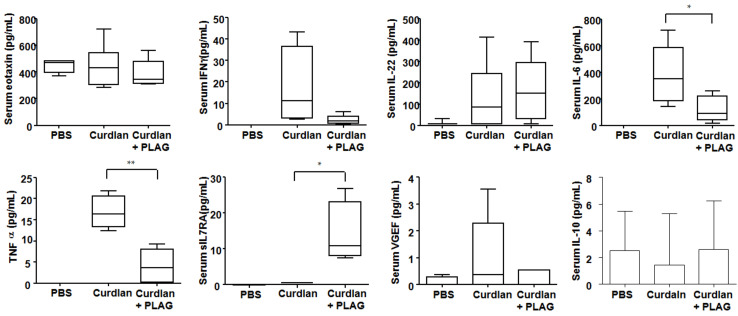
**Serum cytokine levels at 20 weeks after a PBS or curdlan injection in SKG mice.** The PLAG-treated curdlan-induced SKG mice had lower serum IL-6 and TNF-α levels compared with the curdlan-induced SKG mice without PLAG treatment. Interestingly, PLAG treatment upregulated sIL-7Rα (an anti-fibrotic molecule) in the curdlan-induced SKG mice. The values represent the mean of three independent experiments ± SEM. * *p* < 0.05; ** *p* < 0.01. PLAG: acetylated diacylglycerol 1-palmitoyl-2-linoleoyl-3-acetyl-rac-glycerol.

At 20 weeks after the first curdlan injection, the level of serum molecules related to inflammation, neutrophil chemotaxis and fibrosis was observed, as shown in Figure 3. The PLAG-treated mice had lower serum IL-6 and TNF-α levels compared with the curdlan-induced SKG mice without PLAG treatment. Serum eotaxin, IFN-γ, IL-22, VGEF and IL-10 did not differ significantly between these two groups. Interestingly, PLAG treatment in the curdlan-induced SKG mice upregulated sIL-7Rα (an anti-fibrotic molecule).

### 3.4. Citrullinated Histone 3 Expression (NETosis) in the Lung Tissue after PLAG Treatment

**Figure 4:**
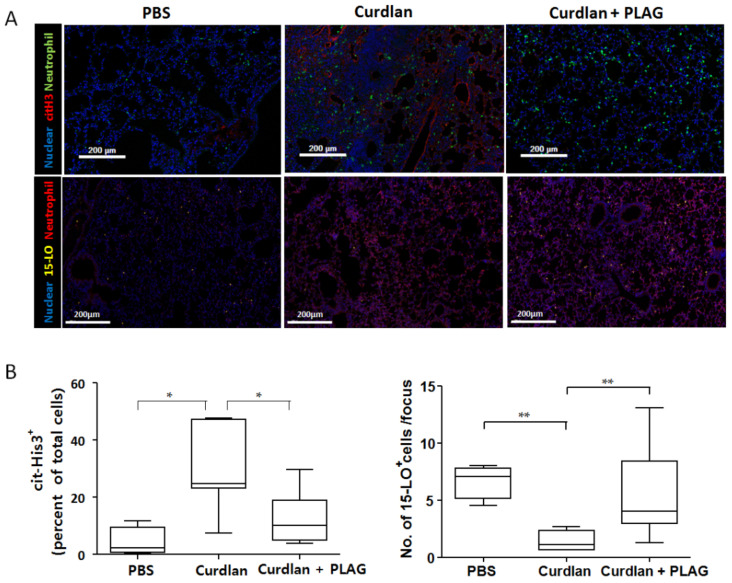
**Opal multiplexed immunofluorescentimages and semi-quantitation of the citrullinated histone 3 and 15-lipoxygenase expression in the lung tissue.** (**A**) Opal multiplexed immunofluorescent images showed the citrullinated histone 3^+^ cells and 15-lipoxygenase^+^ cells in the lung tissue. (**B**) Compared with the curdlan-induced SKG mice, a lower density of citrullinated histone 3^+^ cells and a higher density of 15-lipoxygenase^+^ cells were shown in the curdlan-induced SKG mice with PLAG treatment, suggesting the resolution of NETosis. The values represent the mean of three independent experiments ± SEM. * *p* < 0.05; ** *p* < 0.01. cit-His3: citrullinated histone 3; 15-LO: 15-lipoxygenase.

As shown in Figure 4, the NETosis marker (citrullinated histone 3) and 15-lipoxygenase were evaluated at 20 weeks after the first curdlan injection. Our results showed a significant reduction of the citrullinated histone 3 expression (evidence of NETosis) in the lungs of the curdlan-induced SKG mice treated with PLAG compared with those without PLAG treatment even though there was no difference in the neutrophil counts between them. Furthermore, 15-lipoxygenase, a resolving neutrophil marker, was increased in the PLAG-treated curdlan-induced SKG mice compared with those without PLAG treatment, suggesting the resolution of NETosis.

## 4. Discussion

In this study, we evaluated whether PLAG could prevent autoimmune arthritis-associated ILD in a well-defined murine model and observed significantly reduced lung inflammation and fibrosis in mice treated with PLAG. In the curdlan-induced SKG mice treated with PLAG, the gross features of the lung tissue were similar to those of the PBS-injected SKG mice (control mice). In addition, PLAG treatment reduced infiltrating GM-CSF^+^ neutrophils and NETosis in the lung tissue. These findings could indicate that PLAG has a preventative effect on inflammation and the progression of fibrosis in RA-associated ILD.

A previous study showed that autoreactive T cells in SKG mice are important mediators of inflammatory arthritis and interstitial pneumonitis, indicating that pulmonary inflammation might be related, in part, to autoreactive T cells in SKG mice [26]. However, Wakasa-Morimoto et al. reported that T cells are less involved in the late phase of lung inflammation than other inflammatory cells such as neutrophils [26]. Manganelli et al. also showed that neutrophils accumulate in the bronchoalveolar lavage fluid of connective tissue disease-associated ILD patients [14]. Neutrophils are an important mediator of the development of ILD in RA [12,13,14,15,27]. We confirmed that neutrophils, particularly GM-CSF^+^ neutrophils, were increased in the lungs of the curdlan-induced SKG mice.

Neutrophils can damage tissue but can also produce chemotactic factors that increase leukocyte infiltration [28]. Persistent oxidative damage such as the production of excessive reactive oxygen species by neutrophils might cause lung inflammation and fibrosis [29,30]. Moreover, increased neutrophil infiltration occurs in many acute and chronic lung diseases and is related to pathogenic processes that cause lung tissue damage [31,32]. In particular, neutrophils release their DNA into the extracellular space to form neutrophil extracellular traps (NETs), a complex of DNA and neutrophil-derived antimicrobial molecules [33]. NETs entrap and possibly kill bacteria and play major roles in various inflammatory diseases [34]. Previous studies have shown that an excessive formation of NETs might be associated with lung inflammation and fibrosis in connective tissue disease-associated ILD via impaired NET degradation as well as inducing the activation of lung fibroblasts [17,35,36]. In addition, neutrophil-derived NETs, which contain citrullinated histones, have been proposed as a source of autoantigens that cause autoantibody generation in RA patients.

The GM-CSF is one of the first-line defense mechanisms when clearing pathogens and is usually produced locally by alveolar macrophages [37]. In addition, the GM-CSF is involved in the neutrophil accumulation in airways stimulated by IL-17 or TNF-α via its effect on neutrophil recruitment and survival [38]. The GM-CSF primes neutrophils to release NETs in response to other stimuli such as LPS or C5a [39]. In our study, the total neutrophil counts did not differ between the curdlan-induced mice with or without PLAG treatment but lung inflammation, fibrosis and the GM-CSF^+^ neutrophil accumulation were significantly reduced in the PLAG treatment group, which was followed by the restoration of activated NETosis.

One of therapeutic targets in RA-associated ILD is to restore a normal neutrophil function. PLAG inhibits NETosis and could be considered to be a resolvin of activated neutrophils, which promote pathogen clearance and inflammation by releasing pro-inflammatory cytokines and proteases [40]. Neutrophils also can undergo transient lipid class switching by swapping 5-LO expression for 15-LO expression [41]. When this occurs, neutrophils begin synthesizing pro-resolving lipid mediators that downregulate neutrophil recruitment, reprogram macrophage phenotypes, promote macrophage efferocytosis and enhance tissue repair [42]. Although the mechanism of PLAG has not been clearly known so far, several previous studies have suggested the anti-inflammatory mechanism of PLAG. Lee et al. showed that PLAG rapidly improved a lipopolysaccharide-induced acute lung injury in which it was possible to reduce the production of macrophage inflammatory protin-2 (MIP-2), which acts as a chemotaxis of neutrophils, to prevent the excessive recruitment of neutrophils and to produce reactive oxygen species in a shorter time, thereby avoiding unnecessary and harmful inflammatory responses [21]. Ko et al. reported that PLAG reduced a hepatic injury by attenuating the neutrophil migration into tissue through the inhibiting of the IL-8/vascular cell adhesion molecule (VCAM) expression in a mouse experiment [43]. In our study, PLAG treatment induced an increased 15-LO expression in the lung tissue, promoted the resolving activity of neutrophils and downregulated NETosis, which further inhibits lung tissue damage. Therefore, restoring the neutrophil activation would be more beneficial than inhibiting the neutrophil function. 

A previous study by Kim et al. showed that PLAG ameliorates joint inflammation for 5 weeks by inhibiting the IL-6/STAT3 or MIP-2 signaling pathways [20]. However, in our study, the effect of PLAG on peripheral arthritis was only observed during the early stage. The peripheral arthritis scores significantly decreased from 1 week to 8 weeks after PLAG administration. However, a statistical significance was not found after 9 weeks of PLAG treatment. Although it was unclear why the therapeutic effect did not continue, one possible reason could be that the PLAG dosage might be insufficient to control peripheral arthritis in this model.

In conclusion, the present study shows that PLAG exerts an anti-arthritic effect only in the early phase of autoimmune arthritis. However, PLAG showed therapeutic effects on lung inflammation and fibrosis development in the curdlan-induced SKG mice, which was related to the suppression of NETosis and modulation of the GM-CSF^+^ neutrophils in the lung tissue. Based on our results, we conclude that PLAG has a therapeutic potential in the treatment of RA-associated ILD.

## Data Availability

The data that support the study findings are available from the corresponding author upon reasonable request.
